# Extracranial metastasis of brain glioblastoma outside CNS: Pathogenesis revisited

**DOI:** 10.1002/cnr2.1905

**Published:** 2023-10-09

**Authors:** Maher Kurdi, Saleh Baeesa, Fahad Okal, Ahmed K. Bamaga, Eyad Faizo, Amany A. Fathaddin, Alaa Alkhotani, Mohammed M. Karami, Basem Bahakeem

**Affiliations:** ^1^ Department of Pathology, Faculty of Medicine King Abdulaziz University Rabigh Saudi Arabia; ^2^ Department of Neurosciences King Faisal Specialist Hospital and Research Center Jeddah Saudi Arabia; ^3^ Department of Neuroscience, Neurosurgery Section, King Abdulaziz Medical City National Guard Health Affairs Jeddah Saudi Arabia; ^4^ Department of Paediatric, Faculty of Medicine King Abdulaziz University and Hospital Jeddah Saudi Arabia; ^5^ Department of Surgery, Faculty of Medicine University of Tabuk Tabuk Saudi Arabia; ^6^ Department of Pathology, College of Medicine King Saud University Riyadh Saudi Arabia; ^7^ Department of Pathology, College of Medicine Umm Al‐Qura University Mecca Saudi Arabia; ^8^ Department of Clinical Physiology, Faculty of Medicine King Abdulaziz University Jeddah Saudi Arabia; ^9^ Department of Internal Medicine Umm‐Alqura University Mecca Saudi Arabia

**Keywords:** cause, extracranial metastasis, factors, glioblastoma, pathogenesis

## Abstract

**Background:**

The most prevalent malignant tumor of the CNS in adults is glioblastoma. Despite undergoing surgery and chemoradiotherapy, the prognosis remains unfavorable, with a median survival period ranging between 15 and 20 months. The incidence of glioblastoma metastasis outside CNS is uncommon with only 0.4%–2% reported rate, compared to other tumors that exhibit a 10% incidence rate of metastasis to the brain. On average, it takes about 11 months from the time of initial diagnosis for the tumor to spread beyond CNS. Consequently, the prognosis for metastatic glioblastoma is grim, with a 6‐month survival rate following diagnosis.

**Findings:**

The rarity of extracranial metastasis is attributed to the blood–brain barrier and lack of a lymphatic drainage system, although rare cases of hematogenous spread and direct implantation have been reported. The possible mechanisms remain unclear and require further investigation. Risk factors have been widely described, including previous craniotomy or biopsies, ventricular shunting, young age, radiation therapy, prolonged survival time, and tumor recurrence. Due to the lack of understanding about extracranial metastasis of glioblastoma pathogenesis, no effective treatment exists to date. Aggressive chemotherapies are not recommended for metastatic glioblastoma as their side effects may worsen the patient prognosis.

**Conclusion:**

The optimal treatment for extracranial metastasis of glioblastoma requires further investigation with a wide inclusion of patients. This review discusses the possible causes, factors, and underlying mechanisms of glioblastoma metastasis to different organs.

## 1 INTRODUCTION

Glioblastoma is the most prevalent primary malignant central nervous system (CNS) tumor in adults.^1^ The standard treatment approach involves surgical resection followed by radiotherapy and chemotherapy. However, despite these treatment regimens, the patients' outcomes remain unsatisfactory, with a median survival period ranging from 15 to 20 months.^2^, ^3^ Without treatment, only about one‐third of patients manage to survive for 1 year.^4^ The 5th edition of World Health Organization (WHO) of CNS tumors has recently changed the classification of WHO‐Grade 4 astrocytoma. IDH‐mutant astrocytoma is currently considered a unified tumor type and graded as WHO 2–4. The presence of necrosis and/or microvascular proliferation (MVP) indicates grade 4 and is referred to as astrocytoma IDH‐mutant WHO‐Grade 4. IDH‐wildtype astrocytoma, WHO‐Grade 4, is reserved for glioblastoma and typically exhibits necrosis and/or MVP proliferation.^5^, ^6^ Lack of necrosis or MVP with the presence of *TERTp* mutation, *EGFR* amplification or chromosome 7 gain and/or chromosome 10 loss also indicates the diagnosis of glioblastoma.^5^, ^6^

Glioblastoma rarely metastasizes outside the brain, with a reported incidence of approximately 0.4%–2.0%, which is significantly lower than the 10% incidence rate of CNS metastasis from other types of tumors.^3^, ^7^ The rarity of extracranial metastasis is attributed to presence of the blood–brain barrier (BBB), lack of a lymphatic drainage system, and the “short overall survival (OS).”^8^, ^9^, ^10^, ^11^, ^12^ Extracranial metastasis predominantly occurs in adult males without any specific predilection for race or geographical location.^9^ Earlier studies have indicated that the average duration from the initial diagnosis to the extracranial spread of the tumor is approximately 11 months, however the time from the initial diagnosis to vertebral metastasis specifically is around 26 months.^10^, ^11^ As a result, the OS rate for patients diagnosed with metastatic glioblastoma outside the CNS is about 6 months.^10^, ^11^

**FIGURE 1:**
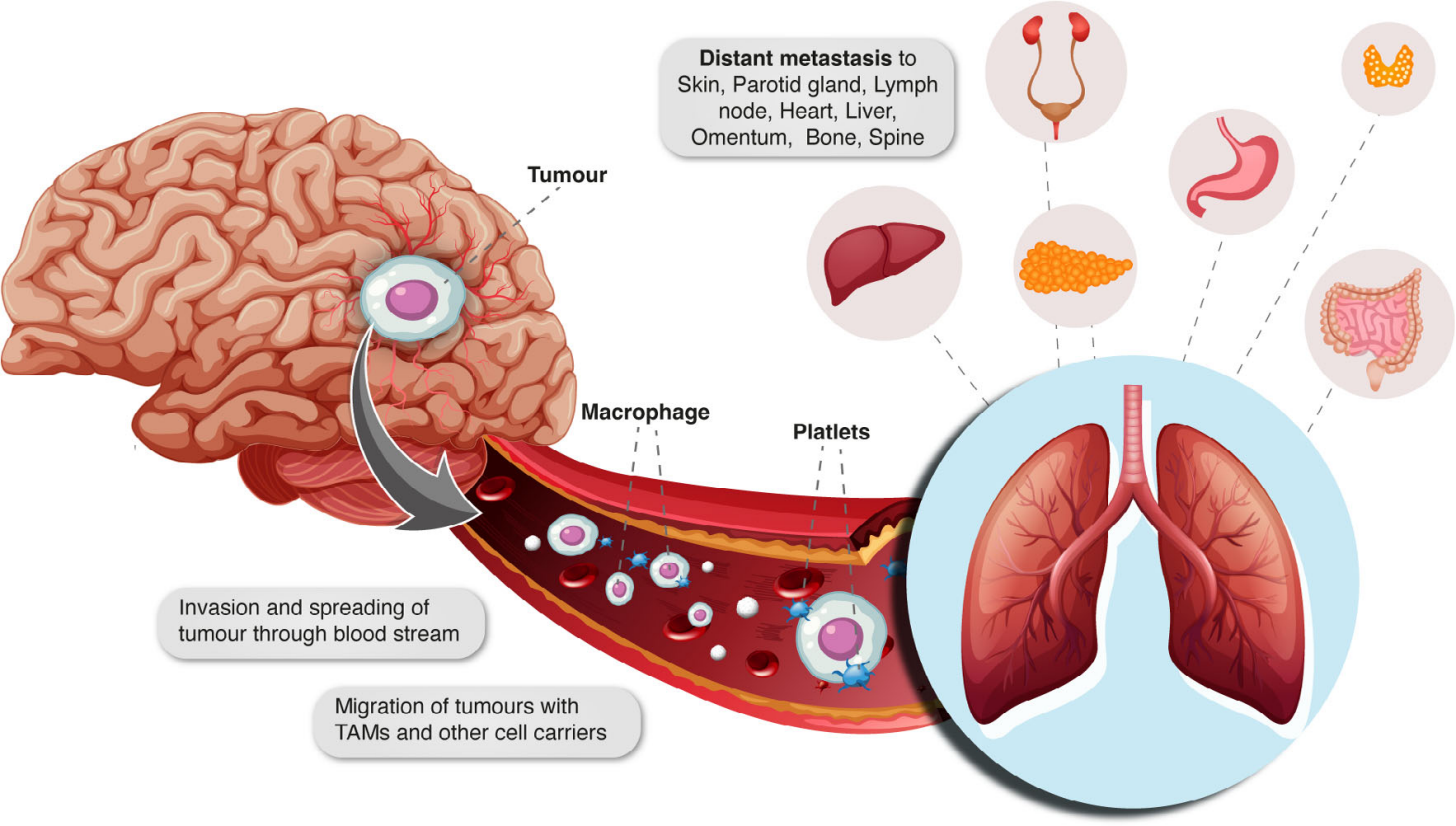
The pathway of extracranial metastasis of glioblastoma to the lung as well as to other distant organs such as liver, heart, kidney, and omentum.

Temporal lobe is the most common primary site for glioblastoma with extracranial metastases, and the spine is the most involved extracranial location near CNS. A meta‐analysis conducted by Cunha and Maldaun,^13^ on 115 cases of metastatic glioblastoma revealed that the majority of cases involved metastases in single organ, with approximately 12 cases exhibiting multi‐site metastases. Common metastatic sites include the lungs and pleura, and lymph nodes followed by liver, skin, scalp, parotid gland, spleen, pancreas, bowel, peritoneum, epidural space, kidney, heart, bones and soft tissue, while metastasis to the meninges or spinal cord via cerebrospinal fluid (CSF) is also frequent^8^, ^14^, ^15^ (Table 1). Among all extracranial metastases, patients with lung metastasis showed the worst prognosis^15^ (Figure 1). Strong et al.^16^ systematically analyzed 92 studies, case reports, and research on bone metastasis from glioblastoma spanning the years 1959–2021. Among these cases, 63% were found to involve the vertebral column, while the remaining cases were associated with lesions within the skull, sternum, rib cage, and appendicular skeleton. Piccirilli et al.,^14^ have collected about 128 cases of extracranial metastases of glioblastoma. It is noteworthy that metastasis in the scalp or around the surgical site can still occur even after previous administration of local radiotherapy, suggesting that radiation around the surgical scar does not provide protection against future cutaneous or subcutaneous invasion of the tumor.^14^

**TABLE 1:** Some examples of glioblastoma cases metastasize outside CNS.

Paper	Age (years)	Gender	IDH status	Metastatic area	Metastatic duration
Liu et al.^37^	46	Male	IDH wildtype	Scalp/lung	20 months
Semonetti et al.^41^	38	Male	IDH wildtype	Lung	4 years
Karatas et al.^42^	55	Male	Unknown	Lung/spine	5 years
Anghileri et al.^43^	30	Male	IDH wildtype	Spine/lung	7 years
Romero‐Rojas et al.^44^	26	Male	Unknown	Parotid gland/bone	6 months
Zhen et al.^45^	25	Male	Unknown	Bone/LN	2 months
Saad et al.^46^	13	Male	Unknown	Leptomeninges/skin	6 months
Toledano et al.^47^	65	Male	Unknown	Spine	10 months
Mujic et al.^48^	39	Male	Unknown	Bowel/pancreas/lung	2 years
Taha et al.^49^	33	Male	Unknown	Parotid gland	6 months
Ogungbu et al.^50^	49	Female	Unknown	Parotid gland/lung	1 years
Bauchesne et al.^51^	54	Male	Unknown	Bone/lung/heart	8 months
Blume et al.^25^	40	Male	IDH mutant	Spine/lung	3 years
Hsu E et al.^52^	53	Female	IDH wildtype	Bone	15 months
Terheggen et al.^20^	12	Male	Unknown	Bone	5 months
Umphlett et al.^36^	74	Female	IDH wildtype	Bone/breast/lung/liver	1 months
Alsardi et al.^53^	43	Female	Unknown	Lung	5 years
Hendrych et al.^37^	43	Female	IDH wildtype	Spine	5 months
Matsuhashi et al.^54^	21	Male	IDH wildtype	Spine	11 months
Nakib et al.^55^	53	Male	IDH wildtype	Skin	6 months
Ghous et al.^31^	65	Male	IDH wildtype	Liver	5 months
Kalokhe et al.^56^	72	Male	Unknown	Lung/rip	10 months
Kalokhe et al.^56^	31	Female	Unknown	Spine	4 months
Zhang et al.^57^	49	Male	IDH‐wildtype	Rip. Spine	24 months
Seo et al.^58^	46	Male	Unknown	Cervical lymph node	12 months

Typically, computed tomography (CT) or magnetic resonance imaging (MRI) of the head and neck is not commonly used as a screening tool for patients diagnosed with glioblastoma. Radiological imaging is recommended when the glioblastoma recurs multiple times, there is prolonged disease progression, or the patient experiences extracranial symptoms. If invasive tumor biopsy or surgery is not an option, liquid biopsy of circulating tumor cells or peripheral blood biomarkers may be considered, although imaging may be less effective than biopsy. Detection of GFAP in liquid biopsy is an obsolete historical tool due to low specificity and the much better sensitivity offered by Polymerase Chain Reaction (PCR) method looking for tumor specific DNA/RNA fragments.^17^ However, the sensitivity and specificity of liquid biopsy need to be improved before its clinical application.^17^

## 2 MECHANISM AND PATHOGENESIS

The possible mechanisms of extracranial metastasis of glioblastoma outside CNS likely include iatrogenic, genetic and molecular factors; however, the cause remains unclear and require further investigation. Various risk factors for extracranial metastasis have been extensively described, including a history of craniotomy, stereotactic biopsies, ventricular shunting, young age, radiation therapy, prolonged survival time, tumor recurrence, and the presence of a sarcomatous component.^18^, ^19^ Hamilton et al.^3^ suggested that metastatic glioblastoma outside CNS are commonly found in patients with prior invasive surgery or biopsy, which could create an iatrogenic access to extracranial structures. Nearly more than 90% of reported patients with extracranial metastases underwent craniotomy beforehand.^19^ Therefore, spreading of glial cells through blood stream during surgery seems to be most likely access.^20^ It is also very rare that patients treated without any surgery develop extracranial metastases.^21^ Noch et al.^22^ examined 10 patients diagnosed with extracranial metastasis of glioblastoma between 2003 and 2007. They found that the median OS from the time of diagnosis was 19 months, and the most common location of metastasis was to the bone, although metastases were also observed in lymph nodes, dura, liver, lung, and soft tissues. They also suggested that risk factors associated with the metastatic cases were including sarcomatous dedifferentiation, disruption of anatomic barriers during surgical resection, and gene mutations.

Glioblastoma metastasis can also occur through the migration of glial cells in the CSF via peritoneal shunt or cavity, or through direct seeding to soft tissues via craniotomy defects.^23^, ^24^ The presence of a chronic wound infection and tumor resection may increase the likelihood of developing extracranial metastasis, possibly due to direct surgical seeding. Conversely, the wound revision procedure itself may enable the hematogenous pathway for metastatic spread.^23^ Nduom et al. and Rong et al. suggested that extracranial metastasis of glioblastoma occurs because of breakdown of the BBB.^25^ Although this mechanism was not extensively studied before, Nduom et al., has described the disruption of endothelial tight junctions and astrocyte–endothelial cell interactions with the breakdown of the blood–brain barrier, which affects peritumoral edema, tumor development, and progression.^25^, ^26^ The nonenhancing areas were associated with preservation of the normal astrocyte–endothelial cell relationship of the preserved BBB.^25^

Cancer cells can spread to remote areas through various mechanisms, such as vascular invasion, lymphatic spread, cranial nerve perineural spread, and direct invasion.^27^ These mechanisms were also observed in glioblastoma. However, there is uncertainty regarding the transmission of malignant glial cells through the bloodstream to other parts of the body. This transmission is considered a hematogenous route. The intracerebral glioblastoma network is distinct and recognizable, with thick‐walled neoplastic vessels composed of multiple layers of endothelial cells that form irregular interconnected glomerular structures arranged in a chaotic manner. Vascular Endothelial Growth Factor (VEGF) is the predominant growth factor in glioblastoma, with concentrations up to 50 times higher in the CNS of patients with this disease than in healthy individuals.^13^ Hence, unlike other systemic malignancies, glioblastoma cells rarely spread through the bloodstream, making hematogenous spread a rare occurrence.^13^

Recently, liquid biopsy is considered as a potential test to detect circulating tumor cells DNA or RNA by analyzing circulating blood or CSF.^28^ However, further studies are needed to validate this method. Liquid biopsy offers several advantages, such as distinguishing tumoral pseudoprogression, selecting targeted therapies, and monitoring the mechanisms of resistance to cytotoxicity and therapeutic targets.

In the case of cervical vertebral metastasis, tumor cells can enter the Batson plexus and spread through the CSF. Additionally, there may be a connection between the meningeal and craniocervical venous systems, which can join the internal vertebral venous plexus.^29^ The mechanisms responsible for osteolytic metastasis of glioblastoma may involve bidirectional interactions between brain tumor cells and bone.^30^ In 2015, two groundbreaking discoveries were made in the field of brain physiology and anatomy: the CNS glymphatic system and the CNS (dural) lymphatic system.^31^ Based on these discoveries, it is likely that brain parenchymal CSF permeates into the glymphatic system, which is then connected with the meningeal lymphatic system. The meningeal lymphatic system is responsible for draining CSF to dural lymphatic vessels. Significantly, the dural lymphatic vessels can transport CSF, CNS antigens, and immune cells to the deep cervical lymph nodes.^31^ Based on the “seed and soil” hypothesis, certain tumor cells have a tendency to metastasize to specific regions within an organ.^22^, ^23^ This suggests that the tumor cells either require a similar microenvironment to grow or possess surface markers that specifically bind to receptors on organ‐ or site‐specific endothelial cells.

Although the molecular variants linked to glioblastoma and its subtypes are well‐documented, a significant knowledge gap exists regarding the genomic drivers that may cause glioblastoma to metastasize.^32^ To evaluate the molecular phenotype of the primary, recurrent, and metastatic lesions, next generation‐sequencing (NGS) panel must be employed. NGS technique can be performed through different methodologies and platforms screening wide range of genetic mutations. Next‐generation sequencing (NGS) technology has revolutionized molecular profiling in cancer research and clinical practices like molecular tumor boards. NGS panels allow for the simultaneous analysis of multiple genes, providing a comprehensive view of the genetic landscape of tumors. This enables identification of specific mutations, gene fusions, copy number alterations, and other genomic alterations that may be driving tumor growth and progression. It also helps identify actionable mutations and genetic alterations that can guide personalized treatment strategies. It enables the selection of targeted therapies or enrollment in clinical trials that specifically target genetic abnormalities, increasing the chances of therapeutic success. The information helps clinicians understand the complexity of the tumor and select appropriate treatment strategies that target the dominant subclones. Specific mutations or gene expression patterns may indicate a higher risk of recurrence, response to specific therapies, or overall prognosis. NGS results can be integrated into multidisciplinary molecular tumor boards, enabling collaboration among pathologists, oncologists, geneticists, and other experts.^33^

It was previously suggested that the metastatic potential of glioblastoma might be related to TP53 gene mutations and the emergence of neoplastic subclones.^34^ Tumor progression may be also facilitated by the overexpression of insulin‐like growth factor binding protein‐1 (IGFBP2) and functional deficiencies in DNA‐dependent protein kinase proteins.^35^ Studies have shown that glioblastomas with extracranial metastasis have higher levels of matrix metalloproteinase than those without extracranial spread.^23^ Umphlete et al.^36^ screened cases with a metastatic glioblastoma to distant sites including breast, liver, and omentum. They found some cases are associated with BRCA1 and ARID1A gene mutations. In The Cancer Genome Atlas (TCGA), glioblastoma specimens showed low occurrence rates of BRCA1 and BRCA2 missense mutations, each at 1.4%.^36^ Newly diagnosed glioblastoma cases have also demonstrated a rare incidence rate of 0.7% for ARID1A mutations, which may be linked to an aggressive phenotype.^36^ Single nucleotide variant in PIK3CA and SMARCB1, has also been identified, predominantly in omental deposits.^36^ Hendrych et al.^37^ has detected NF1, NOTCH3, AIRDA1, and MTOR genetic alteration in metastatic glioblastoma to spine. It was discovered that BRAF mutation is commonly present in the primary tumor but is absent in the metastasis, where NF1 gene mutations are instead detected. This indicates that a subset of tumor cells that lack BRAF mutation may have gained the potential to metastasize.^37^

Up to date, there are no effective treatments for extracranial metastasis of glioblastoma. Single report has indicated that aggressive therapy is not suggested in metastatic glioblastoma because of poor prognosis.^38^ Further research is needed to determine the best treatment approach for extracranial metastasis of glioblastoma, with a wider range of patients included in studies. In certain cases of metastatic glioblastoma with specific gene mutations, immunotherapy may be a viable alternative to temozolomide. Recent findings suggest that PARP inhibitor therapy may be effective for tumors with ARID1A and BRCA1 defects.^39^ Early phase clinical trials are currently assessing the poly (ADP‐ribose) polymerase (PARP inhibitor) as a radio‐ and chemo‐sensitizer for glioblastoma. However, no molecular biomarkers have yet been identified for predicting response.^40^

## 3 CONCLUSION

Metastatic Grade 4 astrocytoma (IDH‐mutant or IDH‐wildtype glioblastoma) outside the CNS is a rare complication. Other than BBB breakdown, possible mechanisms include the migration of glial cells through a peritoneal shunt or cavity, direct seeding, and chronic wound infection. Hematogenous spread is unlikely. Genomic drivers leading to glioblastoma metastasis are not fully understood, but mutations in TP53, IGFBP2, BRACA1, ARID1A, SMARCB1 and matrix metalloproteinase have been associated with tumor progression and dissemination. NGS can assist in evaluating the molecular phenotype and identifying genetic alterations associated with metastatic glioblastoma. Further studies are required to segregate the recent change in grade 4 astrocytoma in term of diagnosis and prognosis.

## AUTHOR CONTRIBUTIONS

**Maher Kurdi:** Conceptualization (lead); project administration (lead); supervision (equal); writing – original draft (equal); writing – review and editing (equal). **Eyad Faizo:** Supervision (equal); writing – original draft (equal); writing – review and editing (equal). **Ahmed K. Bamaga:** Visualization (equal); writing – original draft (equal); writing – review and editing (equal). **Fahad Okal:** Writing – original draft (equal); writing – review and editing (equal). **Saleh Baeesa:** Writing – original draft (equal); writing – review and editing (equal). **Amany M. Fathaddin:** Writing – original draft (equal); writing – review and editing (equal). **Alaa Alkhotani:** Writing – original draft (equal); writing – review and editing (equal). **Mohammed M. Karami:** Writing – original draft (equal); writing – review and editing (equal). **Basem Bahakeem:** Writing – original draft (equal); writing – review and editing (equal).

## CONFLICT OF INTEREST STATEMENT

The authors have stated explicitly that there are no conflicts of interest in connection with this article.

## ETHICS STATEMENT

None.

## INFORMED CONSENT

None.

## Data Availability

The data supporting the findings of this review study are available on request from corresponding author.
